# Pharmacokinetics and Safety of Ceftazidime-Avibactam in Neonates and Young Infants: A Phase 2a, Multicenter Prospective Trial

**DOI:** 10.1093/jpids/piaf028

**Published:** 2025-04-19

**Authors:** John Bradley, Emmanuel Roilides, Margaret Tawadrous, Jean Li Yan, Elena Soto, Gregory G Stone, Shweta Kamat, Paurus Irani, Richard England

**Affiliations:** Department of Pediatrics, Rady Children’s Hospital, University of California San Diego School of Medicine, San Diego, CA, USA; 3^rd^ Department of Pediatrics, Aristotle University and Hippokration Hospital, Thessaloniki, Greece; Research and Development, Pfizer Inc., Groton, CT, USA; Research and Development, Pfizer Inc., Cambridge, MA, USA; Pharmacometrics and Systems Pharmacology, Pfizer Research and Development, Pfizer Inc., Sandwich, Kent, UK; Research and Development, Pfizer Inc., Groton, CT, USA; Global Medical Affairs, Pfizer India Ltd., Mumbai, India; Global Medical Affairs, Pfizer UK Ltd., Tadworth, Surrey, UK; Research and Development, Pfizer Inc., Groton, CT, USA

**Keywords:** neonatal infection, Gram-negative bacteria, antibiotic therapy, antimicrobial resistance, sepsis

## Abstract

**Background:**

This phase 2a study evaluated pharmacokinetics and safety of ceftazidime-avibactam (CAZ/AVI; combination dosed as fixed 4:1 ratio) in neonates and young infants with suspected/confirmed infections due to Gram-negative pathogens requiring intravenous antibiotics.

**Methods:**

Hospitalized neonates and infants (gestational age ≥ 26 weeks to < 3 months), enrolled sequentially into 3 age cohorts, received CAZ/AVI single dose (Part A) or multiple dose every 8 h (Part B) by 2-h intravenous infusions. Infants > 28 days (Cohort 1) received CAZ/AVI 37.5 mg/kg/dose (CAZ 30 mg/kg and AVI 7.5 mg/kg). Full-term neonates ≤ 28 days (Cohort 2) and preterm neonates ≤ 28 days (Cohort 3) received 25 mg/kg/dose (CAZ 20 mg/kg and AVI 5 mg/kg). Pharmacokinetics, safety, and clinical and microbiological outcomes (Part B only) were assessed descriptively.

**Results:**

Forty-six patients received CAZ/AVI, 25 in Part A and 21 in Part B. Sepsis (39.1%) and urinary tract infection (15.2%) were the predominant diagnoses. Observed drug plasma-concentration time profiles were generally similar across cohorts. Overall, 23 patients (50%) had ≥ 1 adverse event (AE), 8 patients (17.4%) had ≥ 1 serious AE (SAE), and 2 patients (4.3%) died; no SAE or death was treatment related. In Part B, ≥ 80% of patients had favorable clinical and microbiological responses.

**Conclusions:**

Plasma exposures after single and multiple CAZ/AVI doses in neonates and young infants < 3 months old (37.5 [30/7.5] mg/kg/dose for > 28 days; 25 [20/5] mg/kg/dose for ≤ 28 days) were similar to approved doses for older children. The safety profile of CAZ/AVI was as expected based on previous observations.

**Study funded by Pfizer. Trial registration**: NCT04126031.

## INTRODUCTION

Globally, neonatal bacterial infections are a leading cause of morbidity and mortality, associated with an estimated 600,000 deaths per year, of which sepsis accounts for almost half (273,000 deaths).^1^ Estimates of the global incidence of neonatal sepsis vary between 3 and 6 million per year, with mortality rates of 11%-19%.^2,3^ Gram-negative bacteria, including Enterobacterales and *Pseudomonas aeruginosa*, are a common cause of infections in healthcare settings, including in neonates.^4–6^ Multidrug-resistant Gram-negative bacteria are an important driver of the increasing prevalence and global burden of antimicrobial resistance.^7–9^ β-lactams, including cephalosporins and carbapenems, remain the mainstay of treatment for serious Gram-negative infections, including in neonates.^10^ However, the effectiveness of these treatments is undermined by the increasing prevalence of extended-spectrum β-lactamase (ESBL)-producing Enterobacterales and carbapenem-resistant Enterobacterales (CRE), which have been identified by the World Health Organization as a critical priority requiring alternative treatment options.^11–14^

Ceftazidime-avibactam (CAZ/AVI), a fixed dose (4:1 ratio) β-lactam/β-lactamase inhibitor combination, was developed to address infections caused by ESBL- and serine carbapenemase-producing Gram-negative bacteria. It has demonstrated efficacy in a range of serious infections in hospitalized adults^15-21^ and children,^22,23^ including those caused by certain CRE, and has a safety profile similar to that of ceftazidime alone.^24^ In adults, CAZ/AVI has been shown to be effective against complicated intra-abdominal infections (cIAIs), complicated urinary tract infections (cUTIs), and hospital-acquired and ventilator-associated pneumonia caused by multidrug-resistant and ESBL-producing Enterobacterales and *P. aeruginosa*,^25,26^ and in patients with bloodstream infections.^27,28^

Ceftazidime monotherapy has been used in children, including neonates, since the 1980s.^29^ CAZ/AVI was approved for children ≥ 3 months old in 2019; however, approved dose recommendations for neonates and infants from birth to < 3 months old were unavailable until 2024 since no pharmacokinetic data for avibactam for this age group had been obtained. The long-established role of ceftazidime in the neonatal setting, alongside the available pediatric data for CAZ/AVI,^22,23^ provide a rationale for extending the evaluation of CAZ/AVI into patients < 3 months old. Population pharmacokinetic modeling using data from the adult and pediatric CAZ/AVI clinical trial programs has established weight-based dosing regimens for pediatric patients ≥ 3 months old that support a high probability of pharmacodynamic target attainment (PTA) against target pathogens.^30^ This phase 2a prospective, open-label, two-part, non-randomized, multicenter study (NCT04126031) evaluated the pharmacokinetics and safety of single and multiple doses of CAZ/AVI in neonates and infants < 3 months old.

## METHODS

### Study Design

The study consisted of single-dose (Part A) and multiple-dose (Part B) administrations of intravenous CAZ/AVI in 3 age cohorts enrolled sequentially by decreasing age (see the Supplementary Methods for design schematic, sequence of cohort startup, and ethical conduct of the study).

### Patients and Treatments

Eligible patients were neonates and infants < 3 months old with suspected or confirmed infections due to Gram‑negative pathogens requiring intravenous antibiotic treatment (see the Supplementary Methods for full inclusion/exclusion criteria). Cohort 1 included full-term infants (gestational age ≥ 37 weeks) with chronological age > 28 days to < 3 months (< 89 days) or preterm infants (gestational age ≥ 26 to < 37 weeks) with corrected age > 28 days to < 3 months (< 89 days). Cohort 2 included full-term neonates (gestational age ≥ 37 weeks), with chronological age from birth to ≤ 28 days. Cohort 3 included preterm neonates (gestational age ≥ 26 to < 37 weeks) from birth to chronological age ≤ 28 days.

In Part A, patients who were already receiving other intravenous antibiotics for suspected or confirmed bacterial infection received a single dose of CAZ/AVI. In Part B, patients with suspected or confirmed bacterial infection with aerobic Gram-negative organisms received 2-14 days of CAZ/AVI treatment, with the potential to switch to oral antibiotics or outpatient parenteral antibiotic therapy after 48 h (see Supplementary Methods).

CAZ/AVI doses were ceftazidime 30 mg/kg/dose and avibactam 7.5 mg/kg/dose for Cohort 1 and ceftazidime 20 mg/kg/dose and avibactam 5 mg/kg/dose for Cohorts 2 and 3, administered by 2-h (± 10 min) intravenous infusion (infusion volume ≤ 2 mL/kg/dose). Patients in Part B received doses every 8 h for up to 14 days.

### Assessments and Endpoints

In Part A, the primary endpoint was ceftazidime and avibactam plasma concentration by nominal sampling time (3 pharmacokinetic samples per patient were obtained on study day 1). Safety and tolerability including treatment-emergent adverse events (AEs), serious AEs (SAEs), deaths, and laboratory abnormalities were assessed as secondary endpoints.

In Part B, safety and tolerability were assessed as primary endpoints. Secondary endpoints comprised ceftazidime and avibactam plasma concentration by nominal sampling time (3 pharmacokinetic samples per patient were obtained on study days 2-14); all-cause mortality and clinical outcome at the end of intravenous treatment (EOIV), end of (all) antibiotic treatment (EOT), a test of cure (TOC) visit 7-14 days after last dose of antibacterial therapy (intravenous or oral), and a late follow-up (LFU) visit 28-35 days after last dose of CAZ/AVI; microbiological response at TOC; and newly emergent infections that were documented after the start of study drug.

### Statistical Methods

No formal hypothesis testing was planned, and the study was not powered for inferential statistical analysis. Descriptive methods were used for all data summaries. Definitions of the study analysis sets are provided in the Supplementary Methods.

## RESULTS

### Patients

**Figure 1.**
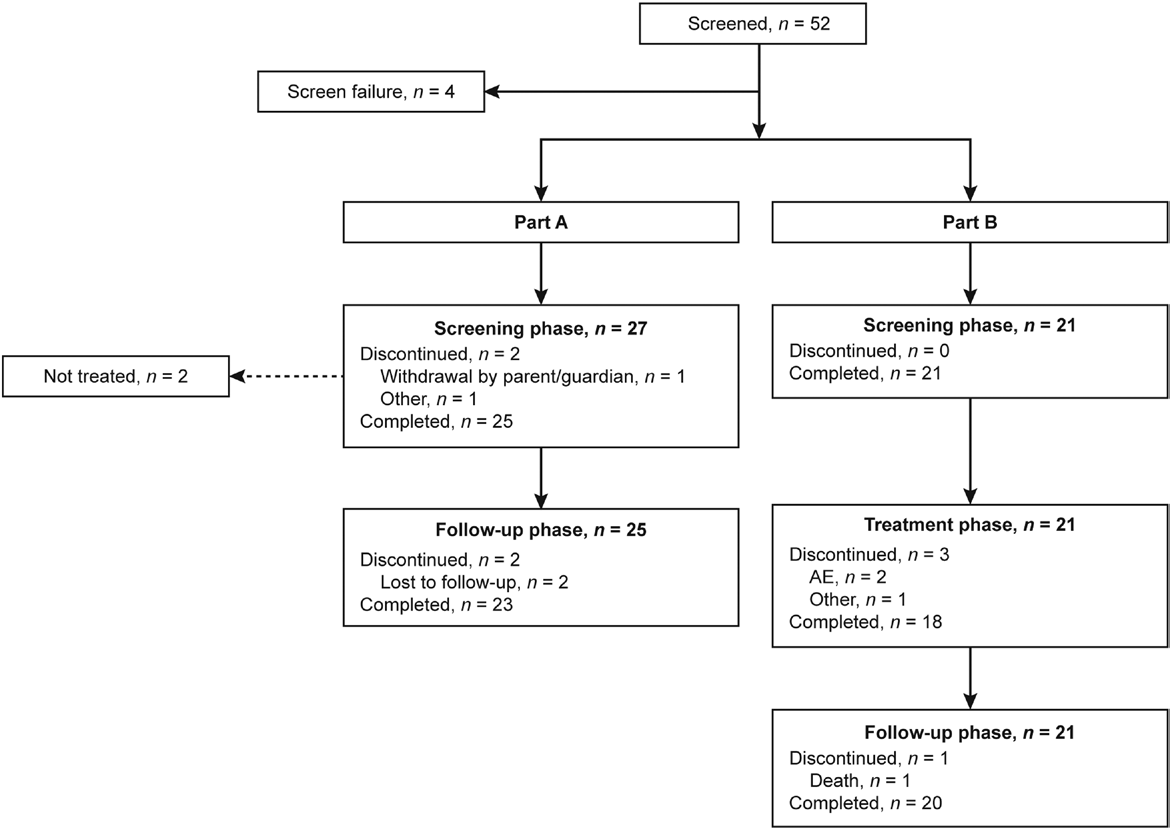
Patient Disposition. In Part A, 2 enrolled patients discontinued from the screening phase prior to receiving study treatment: 1 patient due to withdrawal by parent/guardian and 1 patient due to “other” (parents wished to discontinue because the vein catheter was not working). In Part B, 1 patient (Cohort 2) was discontinued from the treatment phase after receiving 1 dose of study treatment due to sudden onset hypertransaminasemia which was attributed to a previous surgical procedure (of note, the blood sample that indicated hypertransaminasemia was taken prior to administration of the first dose of CAZ/AVI). The investigator decided to continue treatment with commercial CAZ/AVI at the protocol-specified dose, but with the patient excluded from any pharmacokinetic assessments or analysis of efficacy. Cohort 1: Full-term infants with chronological age > 28 days to < 3 months or preterm infants with corrected age > 28 days to < 3 months. Cohort 2: Full-term neonates with chronological age from birth to ≤ 28 days. Cohort 3: Preterm neonates from birth to chronological age ≤ 28 days. AE, adverse event; CAZ/AVI, ceftazidime-avibactam.

The study was conducted between January 2020 and December 2022 at 39 sites in Estonia, Greece, Hungary, India, Italy, the Philippines, Slovakia, Taiwan, and the United States. Of 52 screened individuals, 48 were enrolled (intent-to-treat [ITT] analysis set); 27 in Part A and 21 in Part B (Figure 1). Two patients enrolled in Part A did not receive study treatment; the safety analysis set therefore included 46 patients. In Part A, 23 patients completed the study; 2 patients (both in Cohort 3) were lost to follow-up. In Part B, 18 patients completed the study treatment phase, and 20 patients completed the follow-up phase; 3 patients discontinued study treatment due to AEs (unrelated to CAZ/AVI), of whom 2 completed the follow-up phase (Figure 1).

Patients’ baseline and demographic characteristics are summarized in Supplementary Table S1. Overall, sepsis (18 patients [39.1%]) and UTI (7 patients [15.2%]) were the most common infection diagnoses (Supplementary Table S1). Ten patients of 21 in Part B had a qualifying Gram-negative pathogen identified at baseline (all were Enterobacterales) and were included in the microbiological-ITT (micro-ITT) analysis set (see Supplementary Results). No carbapenem-resistant Enterobacterales were identified in enrolled subjects.

### Safety Evaluation

The median (range) duration of CAZ/AVI treatment was 7.0 (1.0–12.0) days in Part B. Overall (Part A and Part B combined), 23 of 46 patients (50.0%) experienced ≥ 1 all-causality AE up to the LFU visit (Table 1); the most frequent, reported in ≥ 3 patients (≥ 5%) were sepsis (unrelated to CAZ/AVI and mainly reflecting the initial diagnosis), anemia, and vomiting (Supplementary Table S2). Most AEs were mild or moderate in severity; 5 of 46 patients (10.9%) experienced severe AEs. Overall, 1 AE of oxygen saturation decreased in 1 patient (Part A, Cohort 3), which was rated as mild by the investigator, was considered related to study treatment. The event started on study day 1 and resolved without intervention within < 24 h. Eight patients (17.4%) experienced ≥ 1 SAE (Supplementary Table S3), all of which were considered unrelated to study intervention. Necrotizing colitis (2 patients [4.3%]) was the only individual SAE reported in > 1 patient. Grouped by the Medical Dictionary for Regulatory Activities system organ class (SoC), the SoC with the most SAEs was infections and infestations, which included 7 events in 5 patients (10.9%), 6 of which were reported as sepsis or septic shock. Two SAEs resulted in patient deaths (both in Part B), 1 during the study and 1 after study completion; both were considered unrelated to study treatment (see the Supplementary Results for SAE narratives).

**Table 1.** Overview of AEs up to the LFU Visit (Safety Analysis Set)

Patients, *n* (%)	Part A			Part B			Overall
	Cohort 1 (*n* = 9)	Cohort 2 (*n* = 8)	Cohort 3 (*n* = 8)	Cohort 1 (*n* = 8)	Cohort 2 (*n* = 5)	Cohort 3 (*n* = 8)	Total (*N* = 46)
Number of AEs	14	7	5	11	11	23	71
Patients with ≥ 1 AE	4 (44.4)	2 (25.0)	2 (25.0)	4 (50.0)	4 (80.0)	7 (87.5)	23 (50.0)
Patients with ≥ 1 SAE	2 (22.2)	0	1 (12.5)	1 (12.5)	2 (40.0)	2 (25.0)	8 (17.4)
Patients with ≥ 1 treatment-related AE	0	0	1 (12.5)	0	0	0	1 (2.2)
Patients with ≥ 1 severe AE	1 (11.1)	0	0	1 (12.5)	1 (20.0)	2 (25.0)	5 (10.9)
Patients discontinued from study due to AEs^a^	0	0	0	0	0	2 (25.0)	2 (4.3)
Patients discontinued study drug due to AE and continued study^b^	0	0	0	0	0	0	0
Patients with dose reduced or temporary discontinuation due to AE	0	0	0	0	0	0	0

^a^Patients who have an AE record that indicates that the AE caused the patient to be discontinued from the study.

^b^Patients who have an AE record that indicates “Action Drug Withdrawn” but AE did not cause the patient to be discontinued from the study.

Cohort 1: Full-term infants with chronological age >28 days to <3 months or preterm infants with corrected age > 28 days to < 3 months.

Cohort 2: Full-term neonates with chronological age from birth to ≤ 28 days.

Cohort 3: Preterm neonates from birth to chronological age ≤ 28 days.

Abbreviations: AE, adverse event; LFU, late follow-up; SAE, serious adverse event.

In Part A, 8 of 25 patients (32.0%) experienced ≥ 1 AE, 1 patient (4.0%) experienced severe AEs, and 3 patients (12.0%) experienced SAEs (Table 1). In Part B, 15 of 21 patients (71.4%) experienced ≥ 1 AE, 4 patients (19.0%) experienced severe AEs, and 5 patients (23.8%) experienced SAEs (including 2 SAEs that resulted in death; see Table 1 and Supplementary Results). Across Part A and Part B, there were no clinically meaningful trends or concerns in clinical laboratory values. Apart from the single mild treatment-related AE of decreased oxygen saturation (Part A, Cohort 3), there were no clinically meaningful changes in vital signs observations.

### Pharmacokinetic Evaluation

**Figure 2.**
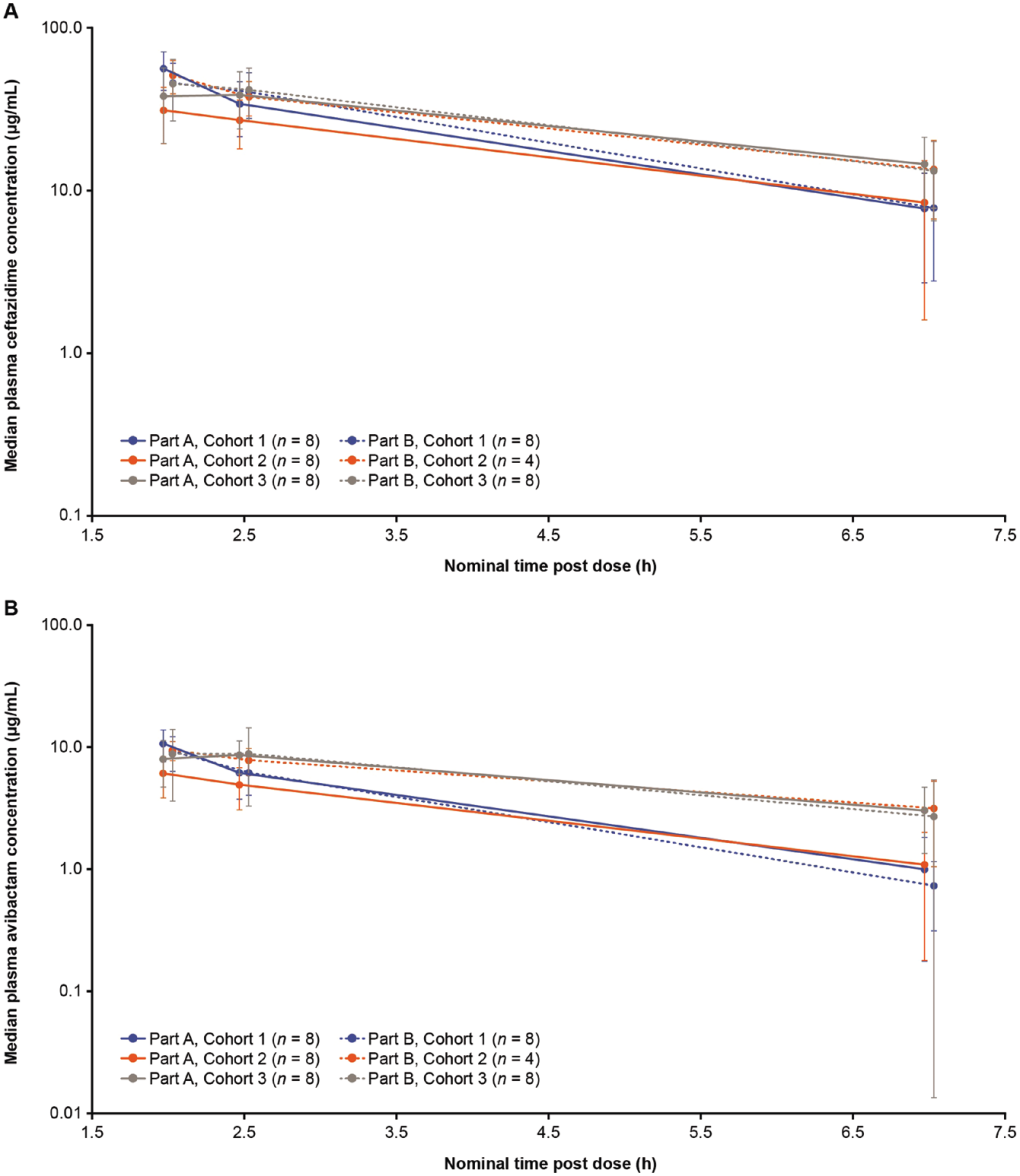
Median Plasma Concentrations of Ceftazidime and Avibactam (Pharmacokinetic Analysis Set, Excludes a Patient with a Medication Error in Part A) Median plasma concentration of (A) ceftazidime and (B) avibactam. Error bars represent % coefficient of variation. Data for Part A and Part B offset around each nominal time point for improved visualization. Cohort 1: Full-term infants with chronological age > 28 days to < 3 months or preterm infants with corrected age > 28 days to < 3 months. Cohort 2: Full-term neonates with chronological age from birth to ≤ 28 days. Cohort 3: Preterm neonates from birth to chronological age ≤ 28 days.

The pharmacokinetic analysis set included 45 patients (25 in Part A and 20 in Part B). Plasma concentrations of ceftazidime and avibactam (Figure 2) were highest within ± 15 min of the end of the 2-h infusion, then gradually decreased as expected at the subsequent sampling time windows (between 0.5 and 1.5 h and between 5 and 6 h after the end of the infusion). At each sampling time point, median plasma concentrations of each drug were generally similar across cohorts in Parts A and B, although in Part A concentrations were slightly lower in Cohort 2 (full-term infants with chronological age from birth to ≤ 28 days) compared to those observed in Cohort 1 (term neonates > 28 days) and Cohort 3 (preterm neonates ≤ 28 days).

A medication error was reported for one patient (Part A, Cohort 1). This patient’s observed plasma ceftazidime and avibactam concentrations were markedly higher than expected, and it was inferred that a documented procedural deviation resulted in an overdose of study treatment. Excluding this patient (Supplementary Tables S4 and S5), median plasma ceftazidime and avibactam concentrations, and coefficients of variation at each collection timepoint were decreased compared with the respective values including the medication error patient.

### Outcomes Evaluation

#### All-Cause Mortality.

As noted above, 2 patients in Part B experienced fatal SAEs, 1 (Cohort 3) on study day 35 (28 days after end of CAZ/AVI treatment) and 1 (Cohort 1) on study day 67 (55 days after end of CAZ/AVI treatment). The all-cause mortality rate for the ITT analysis set, which excluded the patient death on study day 67 as it was outside of the study’s active data collection period, was therefore 4.8% (1 of 21 patients).

#### Clinical Outcomes.

Investigator-assessed clinical outcomes at EOIV, EOT, TOC, and LFU visits (Part B) in the ITT analysis set are shown in Table 2. The proportion of patients with clinical cure at TOC was 81.0% (17 of 21 patients; Table 2). Favorable clinical outcome (cure or improvement) rates of 85.7% (18 of 21 patients) were observed at the EOIV and EOT visits and were sustained through to the LFU visit. One patient (Cohort 3) had clinical failure reported at EOIV due to SAEs leading to discontinuation of study treatment (see the Supplementary Results for SAE narratives); per the study protocol, this failure at EOIV was carried across to all subsequent assessments.

**Table 2.** Clinical Outcomes by Study Visit (Part B; ITT Analysis Set)

Visit	Clinical outcome	Patients, *n* (%)			
		Cohort 1 (*n* = 8)	Cohort 2 (*n* = 5)	Cohort 3 (*n* = 8)	Total (*N* = 21)
EOIV	Favorable outcome	8 (100.0)	4 (80.0)	6 (75.0)	18 (85.7)
	Clinical cure	2 (25.0)	2 (40.0)	3 (37.5)	7 (33.3)
	Clinical improvement	6 (75.0)	2 (40.0)	3 (37.5)	11 (52.4)
	Clinical failure	0	0	1 (12.5)	1 (4.8)
	Indeterminate	0	1 (20.0)	1 (12.5)	2 (9.5)
	Missing	0	0	0	0
EOT	Favorable outcome	8 (100.0)	4 (80.0)	6 (75.0)	18 (85.7)
	Clinical cure	6 (75.0)	3 (60.0)	3 (37.5)	12 (57.1)
	Clinical improvement	2 (25.0)	1 (20.0)	3 (37.5)	6 (28.6)
	Clinical failure	0	0	1 (12.5)	1 (4.8)
	Indeterminate	0	1 (20.0)	0	1 (4.8)
	Missing	0	0	1 (12.5)	1 (4.8)
LFU	Favorable outcome	8 (100.0)	4 (80.0)	6 (75.0)	18 (85.7)
	Clinical cure	8 (100.0)	4 (80.0)	6 (75.0)	18 (85.7)
	Clinical improvement	0	0	0	0
	Clinical failure	0	0	1 (12.5)	1 (4.8)
	Indeterminate	0	1 (20.0)	0	1 (4.8)
	Missing	0	0	0	0
TOC	Favorable outcome	7 (87.5)	3 (60.0)	7 (87.5)	17 (81.0)
	Clinical cure	7 (87.5)	3 (60.0)	7 (87.5)	17 (81.0)
	Clinical improvement	0	0	0	0
	Clinical failure	0	0	1 (12.5)	1 (4.8)
	Indeterminate	0	2 (40.0)	0	2 (9.5)
	Missing	0	0	0	0

Cohort 1: Full-term infants with chronological age > 28 days to < 3 months or preterm infants with corrected age > 28 days to < 3 months.

Cohort 2: Full-term neonates with chronological age from birth to ≤ 28 days.

Cohort 3: Preterm neonates from birth to chronological age ≤ 28 days.

Abbreviations: EOIV, end of intravenous treatment; EOT, end of treatment; ITT, intent-to-treat; LFU, long-term follow-up; TOC, test of care.

Of 2 patients with indeterminate clinical responses at TOC, one (Cohort 1) did not have a TOC visit, but had a favorable clinical outcome at EOIV and LFU. The other patient (Cohort 2) was receiving an oral antibiotic for an AE of postoperative wound infection. There was no culture-identified pathogen, so this event was not reported as a new infection. The patient had a favorable response at the LFU visit, at which time the AE had resolved, and the oral antibiotic was stopped. In the modified ITT analysis set, favorable clinical outcome rates of ≥ 81.0% were observed at the EOIV, EOT, and LFU visits, with a favorable clinical outcome rate of 75.0% at TOC (Supplementary Table S6).

#### Microbiological Outcomes.

The proportion of patients in Part B with a favorable microbiological response (eradication or presumed eradication) at TOC was 80% (8 of 10 patients) in the micro-ITT analysis set. Favorable per-pathogen microbiological responses at TOC were observed for 88.9% (8 of 9) baseline pathogens in the micro-ITT analysis set (see the Supplementary Results for details of missing/unfavorable microbiological responses).

No emergent infections, defined as either superinfection (a culture-identified pathogen other than a baseline pathogen during the course of active treatment with study therapy requiring alternative antimicrobial therapy) or new infection (culture-identified pathogen other than a baseline pathogen at any time after study treatment has finished requiring alternative antimicrobial therapy) were reported for any patient in the micro-ITT analysis set. Two patients in Part B, Cohort 1 had bacterial pathogens in urine cultures collected by catheterization at TOC (1 with *Enterococcus faecium* [10^4^ CFU/mL] and one with *Klebsiella pneumoniae* [> 10^3^ but < 10^4^ CFU/mL]); however, clinical cure and favorable microbiological responses at TOC were recorded for both patients (neither patient required alternative antimicrobial therapy because of the urine cultures). One patient (Part B, Cohort 1) with a diagnosis of epidermolysis bullosa was enrolled for the treatment of sepsis and experienced severe SAEs of sepsis with *E. cloacae* and *Candida parapsilosis* 11 and 21 days, respectively, after the last dose of CAZ/AVI (see the Supplementary Results for SAE narratives). The patient was admitted to another hospital and the associated microbiological data were not available for analysis.

## DISCUSSION

Regional and international guidelines for managing neonatal infections currently recommend empiric antibiotic treatment of newborns with clinical signs of infection, particularly those with maternal risk factors.^31,32^ Gram-negative bacteria including *E. coli*, *Klebsiella* spp., and *Pseudomonas* spp. are estimated to cause 20%-28% of early-onset infant bacteremia cases,^33,34^ and are also responsible for 20%-35% of late-onset bacteremia cases.^34,35^ Real-world observational data suggest that CAZ/AVI addresses an unmet clinical need by supporting positive clinical outcomes for pediatric patients, including neonates, infected with Enterobacterales and *P. aeruginosa* resistant to many standard-of-care β-lactam antibiotics due to the presence of ESBLs, AmpC, and *K. pneumoniae* carbapenemases.^36^

The current study evaluated CAZ/AVI in infants and premature neonates < 3 months old down to a gestational age of 31 weeks with suspected or confirmed infections due to Gram-negative pathogens requiring intravenous antibiotic treatment. The findings supported the addition of the age- and weight-based neonatal doses used in the study to the approved CAZ/AVI product labeling. The open-label, non-randomized design and the small numbers of patients in individual age cohorts are study limitations. Although our study was designed to assess pharmacokinetics and safety in children receiving standard-of-care antibiotic therapy, no patients with carbapenem-resistant Enterobacterales were identified. The study was designed primarily to evaluate tolerability, safety, and pharmacokinetics, and the numbers of patients were sufficient to fulfill those objectives. The effectiveness of CAZ/AVI has been demonstrated in older pediatric populations.^22,23^ The safety profile of CAZ/AVI in the neonatal population was as expected based on observations in adults and older children.^22-24^ Most reported AEs were mild or moderate in severity and/or consistent with underlying conditions, and no new safety concerns were identified. Two patient deaths were reported (both in Part B), 1 during the study and 1 after study completion; both were due to severe underlying conditions and unrelated to CAZ/AVI.

In Part B, the favorable clinical outcome rate at TOC was 81% (17 of 21 patients) in the ITT analysis set and the favorable microbiological response rate at TOC was 80% (8 of 10 patients) in the micro-ITT analysis set. These results are similar to those reported in older pediatric patients with cIAIs or cUTIs treated with CAZ/AVI in prospective, randomized registration trials.^22,23^ The microbiological response results should be interpreted with additional caution because of the low number of patients with microbiologically confirmed infection (*n* = 10).

The prolonged 2-h CAZ/AVI infusion is designed to maximize the pharmacodynamic property of time that antibiotic concentrations in serum exceed the pathogen minimum inhibitory concentration during a dosing interval, and the safety and effectiveness of CAZ-AVI has been demonstrated in older children^22,23^ and adults.^15-21^ Observed plasma concentration time profiles were as expected and generally similar between cohorts. Following single or multiple intravenous infusions of CAZ/AVI, plasma concentrations were highest within ± 15 min of the end of the 2-h infusion, then gradually decreased. Slightly faster-observed clearance of both drugs was evident in Cohorts 1 and 2, consistent with the older developmental age and more developed renal function of these patients. Excluding an outlier in Part A, plasma ceftazidime and avibactam concentration-time profiles were broadly consistent with observations and dosing simulations in older children.^22,23,30^ A population pharmacokinetic modeling analysis using data from the current study (to be reported separately) will estimate pharmacokinetic parameters in the neonatal population and simulate exposures and PTA in neonates and infants < 3 months old.

In summary, these findings provide supportive evidence for the safety of approved CAZ/AVI doses for premature and full-term neonates and infants < 3 months old (37.5 [30/7.5] mg/kg/dose for > 28 days; 25 [20/5] mg/kg/dose for ≤ 28 days) for the treatment of suspected or confirmed Gram-negative bacterial infections. Plasma drug concentrations were consistent with pharmacokinetic results in older children.

## Supplementary Material

piaf028_suppl_Supplementary_Tables_S1-S6

## Data Availability

Upon request, and subject to review, Pfizer will provide the data that support the findings of this study. Subject to certain criteria, conditions, and exceptions, Pfizer may also provide access to the related individual de-identified participant data. See https://www.pfizer.com/science/clinical-trials/trial-data-and-results for more information.
