# Supplementary material for: Pharmacokinetics and Safety of Ceftazidime-Avibactam in Neonates and Young Infants: A Phase 2a, Multicenter Prospective Trial
Source: J Pediatric Infect Dis Soc. 2025 Apr 19;14(5):piaf028. doi: 10.1093/jpids/piaf028 (PMC12117184; doi:10.1093/jpids/piaf028)
Supplement: piaf028_suppl_Supplementary_Tables_S1-S6 [file piaf028_suppl_supplementary_tables_s1-s6.docx]

Supplementary Appendix

Pharmacokinetics and Safety of Ceftazidime-Avibactam in Neonates and Young Infants: a Phase 2a, Multicenter Prospective Trial

John Bradley,^1^ Emmanuel Roilides,^2^ Margaret Tawadrous,^3^ Jean Li Yan,^4^ Elena Soto,^5^ Gregory G. Stone,^3^ Shweta Kamat,^6^ Paurus Irani,^7^ Richard England^3^

^1^Department of Pediatrics, University of California San Diego School of Medicine/Rady Children’s Hospital, San Diego, CA, USA
^2^3^rd^ Department of Pediatrics, Aristotle University and Hippokration Hospital, Thessaloniki, Greece
^3^Research and Development, Pfizer Inc., Groton, CT, USA
^4^Research and Development, Pfizer Inc., Cambridge, MA, USA
^5^Pharmacometrics and Systems Pharmacology, Pfizer Research and Development, Pfizer Inc., Sandwich, Kent, UK
^6^Global Medical Affairs, Pfizer India Ltd., Mumbai, India
^7^Global Medical Affairs, Pfizer UK Ltd., Tadworth, Surrey, UK

# Supplementary Methods

## Study design


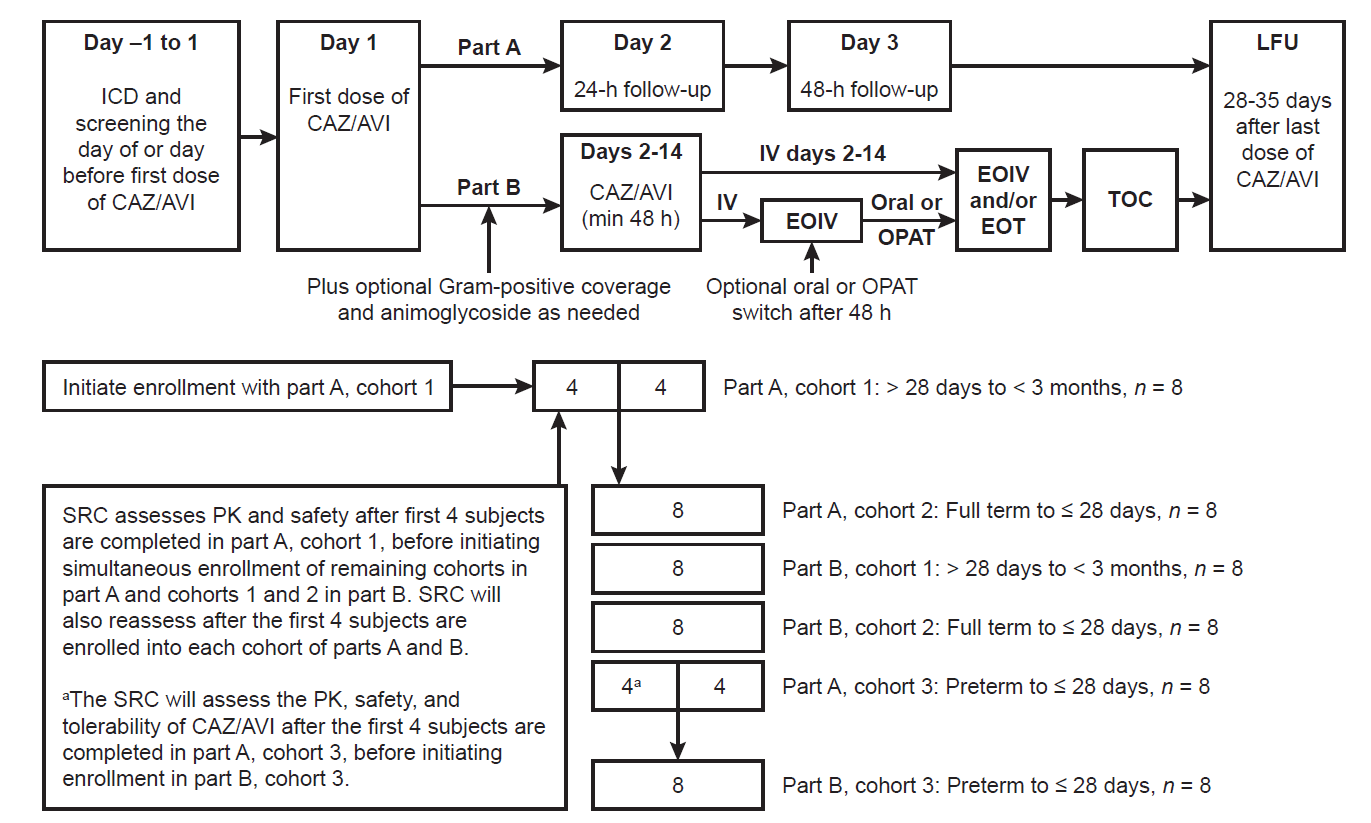


An external Data Monitoring Committee was responsible for ongoing monitoring of the safety of study patients. A sponsor Safety Review Committee (including the international coordinating investigator, J.S.B) assessed PK, safety, and tolerability data from each cohort throughout the study.

Cohort 1: Full-term infants with chronological age > 28 days to < 3 months or preterm infants with corrected age > 28 days to < 3 months.
Cohort 2: Full-term neonates with chronological age from birth to ≤ 28 days.
Cohort 3: Preterm neonates from birth to chronological age ≤ 28 days.

Abbreviations: CAZ/AVI, ceftazidime-avibactam; EOIV, end of intravenous treatment; EOT, end of (all) treatment; h, hours; ICD, informed consent document; IV, intravenous; LFU; late follow-up; OPAT, outpatient parenteral antibiotic therapy; PK, pharmacokinetics; TOC, test of cure.

## Ethics and informed consent

The study protocol, any protocol amendments, and other relevant documents were approved by the participating sites’ national/institutional review boards. All patients parent(s), or their legal guardian(s) signed a statement of informed consent that met the requirements of International Council for Harmonisation of Technical Requirements for Pharmaceuticals for Human Use Good Clinical Practice (ICHGCP), local regulatory requirements, and legal requirements, including applicable privacy laws. The study was conducted in accordance with the protocol and consensus ethical principles derived from international guidelines including the Declaration of Helsinki Council and Council for International Organizations of Medical Sciences International Ethical Guidelines, applicable ICH GCP guidelines, applicable ISO 14155 guidelines, medical device guidelines, and other applicable laws and regulations, including privacy laws.

## Inclusion criteria for all participants

1. Evidence of a personally signed and dated informed consent document indicating that the subject’s parent(s), legal guardian, or legally acceptable representative has been informed of all pertinent aspects of the study.
2. Willing and able to comply with scheduled visits, treatment plan, laboratory tests, and other study procedures.
3. Male or female neonates and infants with age at screening:
   1. Cohort 1: Full-term infants (gestational age ≥ 37 weeks) with chronological age > 28 days to < 3 months (< 89 days) or preterm infants with corrected age > 28 days to < 3 months (< 89 days). A maximum of 3 preterm corrected-age infants could be enrolled in each part (A and B) of Cohort 1. Sites were to be notified in writing if this limit was reached.
   2. Cohort 2: Full-term neonates (gestational age ≥ 37 weeks) from birth to ≤ 28 days.
   3. Cohort 3: Preterm neonates (gestational age ≥ 26 to < 37 weeks) from birth to ≤ 28 days.

Gestational age is the time elapsed between the first day of the last menstrual period and birth.

Corrected age is the age of the infant from the expected date of delivery, calculated by subtracting the number of weeks born before 40 weeks of gestation from the chronological age (Engle 2004) [1]. Corrected age (in weeks) = chronological age in weeks – (40 – gestational age in weeks).

## Inclusion criteria for Part A only

1. Hospitalized and receiving intravenous antibacterial therapy for the treatment of a suspected or confirmed bacterial infection.

## Inclusion criteria for Part B only

1. Hospitalized with a suspected or confirmed aerobic Gram-negative bacterial infection requiring intravenous antibacterial therapy.
2. Patients must meet at least 1 clinical and 1 laboratory criterion or meet at least 2 of the clinical criteria.

Clinical criteria:

1. Hypothermia (< 36°C) OR fever (> 38.5°C);
2. Bradycardia OR tachycardia OR rhythm instability;
3. Urine output 0.5 to 1 mL/kg/h OR hypotension OR mottled skin OR impaired peripheral perfusion;
4. Petechial rash OR sclerema neonatorum;
5. New onset or worsening of apnea episodes OR tachypnea episodes OR increased oxygen requirements OR requirement for ventilation support;
6. Feeding intolerance OR poor suckling OR abdominal distension;
7. Irritability;
8. Lethargy;
9. Hypotonia.

Laboratory criteria:

1. White blood cell count ≤ 4.0 × 10^9^/L OR ≥ 20.0 × 10^9^/L;
2. Immature to total neutrophil ratio > 0.2;
3. Platelet count ≤ 100 × 10^9^/L;
4. C-reactive protein > 15 mg/L OR procalcitonin ≥ 2 ng/mL;
5. Hyperglycemia OR hypoglycemia;
6. Metabolic acidosis.

## Exclusion criteria for all participants

1. Investigator site staff members directly involved in the conduct of the study and their family members; site staff members otherwise supervised by the investigator; or subjects who are Pfizer employees, including their family members, directly involved in the conduct of the study.
2. Participation in another clinical study involving investigational drug(s) within 30 days prior to study entry and/or during this study participation, or have previously participated in the current study or in another study of ceftazidime-avibactam (CAZ/AVI) in which an active agent was received.
3. Use of potent inhibitors of organic anion transporters (OAT) 1 and/or OAT3 (e.g., probenecid, para-aminohippuric acid, or teriflunomide) are prohibited. This prohibition of OAT1 and/or OAT3 inhibitors also applies to the mothers of any neonates or infants who are breastfeeding during the trial.
4. Other acute or chronic, medical or laboratory abnormality that may increase the risk associated with study participation or investigational product administration, or may interfere with the interpretation of study results and, in the judgment of the Investigator, would make the subject inappropriate for entry into this study.
5. Documented history of any hypersensitivity or allergic reaction to any β-lactam antibiotic.
6. Refractory septic shock within 24 h before screening that does not resolve after 60 min of vasopressor therapy.
7. Moderate or severe renal impairment defined as serum creatinine ≥ 2 times the upper limit of normal for age OR urine output < 0.5 mL/kg/h (measured over at least 8 h) OR requirement for dialysis. Deterioration of renal function after enrollment during Part B of the study will be handled on a case-by-case basis in discussion with the Medical Monitor.
8. Evidence of progressively fatal underlying disease, or life expectancy of ≤ 60 days.
9. Documented history of seizure.
10. Active acute viral hepatitis or acute hepatic failure.
11. Known *Clostridium difficile-*associated diarrhea.
12. Requiring or currently taking antiretroviral therapy for human immunodeficiency virus (HIV) or known HIV-positive mother.
13. Any condition (e.g., cystic fibrosis, urea cycle disorders), antepartum/peripartum factors, or procedures that would, in the opinion of the Investigator, make the subject unsuitable for the study, place a subject at risk, or compromise the quality of data.

14. Treatment with ceftazidime within 12 h of CAZ/AVI administration.

## Exclusion criteria for Part A only

1. Subject received a blood or a blood component transfusion within 24 h of the start of CAZ/AVI infusion.
2. Subject is expected to be discharged less than 24 h after the start of CAZ/AVI infusion.

## Exclusion criteria for Part B only

1. At study entry, subject has a confirmed or strongly suspected infection with a pathogen known to be resistant to CAZ/AVI; or only a Gram-positive pathogen; or viral, fungal, or parasitic pathogens as the sole cause of infection.
2. Confirmed or suspected central nervous system infection (e.g., meningitis, brain abscess, subdural abscess).
3. Anticipated need for antibacterial therapy for > 14 days (e.g., osteomyelitis, endocarditis). This applies to both study treatment with CAZ/AVI as well as adjunctive intravenous antibacterial treatment for suspected co-infection with Gram-positive organisms or multidrug-resistant Gram-negative organisms.
4. Receipt of more than 24 h of non-study, systemic, antibacterial treatment for Gram-negative organisms after culture and before administration of study doses of CAZ/AVI. Empiric coverage with an aminoglycoside for suspected multidrug-resistant organisms is permitted, provided that CAZ/AVI is initiated within 24 h after culture.
5. Intravenous treatment with chloramphenicol within 24 h of administration of study doses of CAZ/AVI.
6. Subject is expected to be discharged less than 48 h after the start of CAZ/AVI infusion.

## Oral and outpatient parenteral antibiotic therapy

Patients who were deemed appropriate for hospital discharge, but still required parenteral antibiotic therapy, could not continue to receive CAZ/AVI study drug outside the hospital. If patients were candidates for oral therapy or outpatient parenteral antibiotic therapy (OPAT) after at least 48 h of intravenous CAZ/AVI treatment, investigators selected the appropriate intravenous antibiotic(s) based on pathogen identification and susceptibility, local therapeutic guidelines, and approved drugs in their respective countries.

## Analysis sets definitions


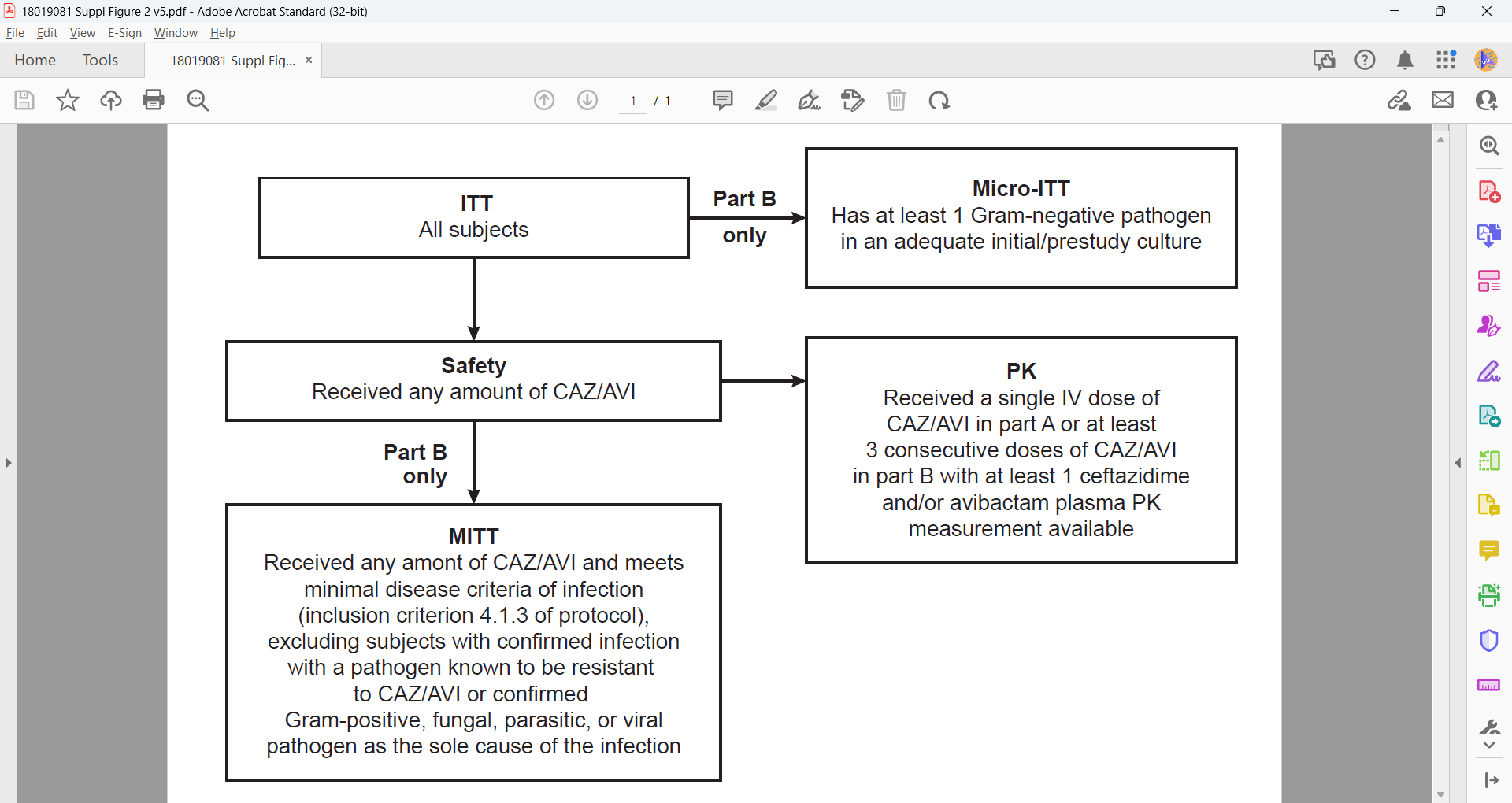


Safety data were summarized for all patients in the safety analysis set. All-cause mortality was summarized for the ITT analysis set (all enrolled patients). Clinical and microbiological outcomes (Part B only) were summarized for the ITT analysis set, MITT analysis set, and micro-ITT analysis set. PK data were summarized by nominal sampling time window for the PK analysis set.

Abbreviations: CAZ/AVI, ceftazidime-avibactam; ITT, intent-to-treat; IV, intravenous; micro-ITT, microbiological intent-to-treat; MITT, modified intent-to-treat; PK, pharmacokinetic.

Supplementary Results

## Baseline pathogens (Part B)

Microbiological assessments (including culture of baseline blood/urine/tissue samples) were performed in Part B only, if clinically indicated as part of patients’ routine care.

All baseline pathogens identified in Part B were Enterobacterales, most commonly *Escherichia coli* (*n* = 6; all in Cohort 1). No baseline pathogens were resistant to CAZ/AVI. The following pathogens were each identified in 1 patient: *Citrobacter koseri* (Cohort 1), *Enterobacter cloacae* complex (Cohort 3), *Klebsiella oxytoca* (Cohort 3), and *K. pneumoniae* (Cohort 3). Two patients had baseline isolates that were resistant to ceftazidime, 1 *E. coli* (Cohort 1) and 1 *K. pneumoniae* (Cohort 3).

## Narratives of serious adverse events (Part A)

In Part A, 8 of 25 patients (32.0%) experienced ≥ 1 adverse event (AE), 1 patient (4.0%) experienced severe AEs, and 3 patients (12.0%) experienced serious AEs (SAEs). Narratives for the 3 patients with SAEs are provided below.

A 7-week-old White female (Part A, Cohort 1) with a primary infectious diagnosis of sepsis and a medical history of hyperbilirubinemia, conjunctivitis, food protein-induced enterocolitis syndrome, diaper dermatitis, alanine aminotransferase (ALT) increased, anemia, hypoalbuminemia, and hyponatremia experienced a severe AE of hyponatremia and a severe SAE of hepatic enzyme increased, both during the follow-up phase. The event of hyponatremia resolved and the event of hepatic enzyme increased was ongoing at the late follow-up (LFU) visit. Both events were considered unrelated to study treatment.

A 5-week-old Black or African American female (Part A, Cohort 3) with a primary infectious diagnosis of necrotizing colitis and a medical history of congenital musculoskeletal disorder of spine, congenital naevus, a double inlet left ventricle, feeding disorder, pulmonary vascular disorder, abnormal magnetic resonance imaging of the head, pulmonary artery banding, hematochezia, agitation, postoperative respiratory failure, and procedural pain experienced an SAE of necrotizing colitis. The condition was also present before study enrollment and was reported as resolved by study day 23.

A 3-week-old White female (Part A, Cohort 3) with a primary infectious diagnosis of necrotizing colitis and a medical history of prematurity (gestational age 31 weeks), premature rupture of membranes, hyperbilirubinemia, and neonatal anemia experienced a moderate SAE of COVID-19 infection during the follow-up phase that was considered unrelated to study treatment. The SAE resolved within 24 days without additional treatment.

## Narratives of serious adverse events (Part B)

In Part B, 15 of 21 patients (71.4%) experienced ≥ 1 AE, 4 patients (19.0%) experienced severe AEs, and 5 patients (23.8%) experienced SAEs (including 2 SAEs that resulted in death). Narratives for the 5 patients with SAEs are provided below.

A 6-week-old Black or African American female (Part B, Cohort 1) with a primary infectious diagnosis of sepsis and a medical history of epidermolysis bullosa, anemia, and hypoalbuminemia received 10 days of intravenous CAZ/AVI treatment. The patient experienced severe SAEs of *Enterobacter* sepsis with positive blood culture for *E. cloacae* (study day 23) and *Candida* sepsis (study day 33). Both events were considered unrelated to study treatment and were ongoing at the LFU visit. The *Candida* sepsis resulted in patient death on study day 67 (outside of the active data collection period). The recurrent infections and death in this patient were believed to be a result of epidermolysis bullosa, a condition known to be associated with mortality at a very young age.

A 2-week-old White male (Part B, Cohort 2) with a primary infectious diagnosis of sepsis and a medical history of hypoglycemia, thrombocytopenia, and retinal hemorrhage received 7 days of intravenous CAZ/AVI treatment. The patient experienced severe SAEs of oliguria and acute respiratory failure, and a moderate SAE of neutropenia during the treatment phase. All 3 events resolved and were considered unrelated to study treatment.

A 2-week-old White male (Part B, Cohort 3) with a primary infectious diagnosis of sepsis and a medical history of prematurity (gestational age 33 weeks), ventricular septal defect, cholestasis, and cardiac failure received 12 days of intravenous CAZ/AVI treatment. The patient experienced severe SAEs of acute cardiac failure and sepsis during the treatment phase that led to discontinuation of the study treatment (this patient was assessed as a clinical failure at end of intravenous treatment [EOIV]). Both events resolved and were considered unrelated to the study treatment.

A 5-week-old (gestational age 34 weeks) White female (Part B, Cohort 3) with a primary infectious diagnosis of sepsis and a medical history of ankle and foot deformities, multiple congenital abnormalities, hypertransaminasemia, cholestatic jaundice, hypoalbuminemia, and hypertrophic cardiomyopathy received 7 days of intravenous CAZ/AVI treatment. The patient experienced a severe SAE of renal failure during the treatment phase, and severe SAEs of necrotizing colitis and septic shock during the follow-up phase. The event of renal failure led to discontinuation of the study treatment. The events of necrotizing colitis and septic shock were fatal. All 3 events were considered unrelated to the study treatment.

A 2-week-old White male (Part B, Cohort 2) with a primary infectious diagnosis of sepsis and a medical history of prematurity (gestational age 37 weeks), ventricular septal defect, cholestasis, and cardiac failure received 10 days of intravenous CAZ/AVI treatment and 18 days of intravenous vancomycin (which was optional per-protocol). Clinical outcome at EOIV, test-of-cure (TOC) and late follow up visits was clinical cure. The patient experienced a mild AE of clonus on study day 2, a moderate SAE of suspected sepsis on study day 24, and a moderate SAE of enterococcal sepsis (*Enterococcus faecium* sepsis) on study day 36 (both sepsis events occurred during the follow-up phase). The initial sepsis SAE was treated with ceftazidime and resolved by study day 32. The enterococcal sepsis was treated with amoxicillin-clavulanate and piperacillin-tazobactam, and was ongoing at the late-follow up visit and eventually resolved on study day 48. All 3 events were considered unrelated to the study treatment.

## Details of missing/unfavorable microbiological responses (Part B)

Two of 10 patients in the micro-ITT analysis set had missing/unfavorable microbiological responses at the TOC visit. One patient (Cohort 1) who had *Citrobacter koseri* isolated from a baseline blood culture did not have a TOC visit, and the per-pathogen microbiological response at TOC was therefore missing for this patient. An unfavorable microbiological response (presumed persistence) at TOC was reported for 1 pathogen (*Enterobacter cloacae* complex) because the patient (Cohort 3) was assessed as a clinical failure at EOIV and switched to other antibiotics, although the baseline pathogen was not culture-verified to be persistent.

Table S1. Patients’ Demographic and Baseline Characteristics (Safety Analysis Set)

| **Variable** | **Part A (*n* = 25)** | **Part B (*n* = 21)** | **Total (*N* = 46)** |
| --- | --- | --- | --- |
| Median (range) chronological age, days^a^ | 25.0 (2, 89) | 23.0 (6, 86) | 23.5 (2, 89) |
| Median (range) corrected age, days^b^ | 45.0 (34, 56) | N/A | 45.0 (34, 56) |
| Median (range) inclusion age, days^c^ | 25.0 (2, 71) | 23.0 (6, 86) | 23.5 (2, 86) |
| Sex, *n* (%) |  |  |  |
| Male | 10 (40.0) | 11 (52.4) | 21 (45.7) |
| Female | 15 (60.0) | 10 (47.6) | 25 (54.3) |
| Race, *n* (%)^d^ |  |  |  |
| White | 18 (72.0) | 18 (85.7) | 36 (78.3) |
| Black or African American | 2 (8.0) | 2 (9.5) | 4 (8.7) |
| Asian | 4 (16.0) | 1 (4.8) | 5 (10.9) |
| American Indian or Alaska Native | 0 | 0 | 0 |
| Native Hawaiian or Other Pacific Islander | 0 | 0 | 0 |
| Not reported | 1 (4.0) | 0 | 1 (2.2) |
| Ethnicity, *n* (%)^d^ |  |  |  |
| Hispanic or Latino | 2 (8.0) | 0 | 2 (4.3) |
| Not Hispanic or Latino | 21 (84.0) | 21 (100.0) | 42 (91.3) |
| Not reported | 2 (8.0) | 0 | 2 (4.3) |
| Primary diagnosis, *n* (%) |  |  |  |
| Bacterial Infection | 5 (20.0) | 3 (14.3) | 8 (17.4) |
| Bacteremia | 1 (4.0) | 1 (4.8) | 2 (4.3) |
| Bacterial infection | 3 (12.0) | 2 (9.5) | 5 (10.9) |
| Enterococcal bacteremia | 1 (4.0) | 0 | 1 (2.2) |
| Infection | 4 (16.0) | 2 (9.5) | 6 (13.0) |
| Enterovirus infection | 1 (4.0) | 0 | 1 (2.2) |
| Escherichia infection | 0 | 1 (4.8) | 1 (2.2) |
| Infection | 1 (4.0) | 0 | 1 (2.2) |
| Neonatal infection | 1 (4.0) | 0 | 1 (2.2) |
| Nosocomial infection | 1 (4.0) | 0 | 1 (2.2) |
| Staphylococcal infection | 0 | 1 (4.8) | 1 (2.2) |
| Leukocytosis | 0 | 1 (4.8) | 1 (2.2) |
| Leukocytosis | 0 | 1 (4.8) | 1 (2.2) |
| Necrotizing colitis | 3 (12.0) | 0 | 3 (6.5) |
| Necrotizing colitis | 3 (12.0) | 0 | 3 (6.5) |
| Sepsis | 8 (32.0) | 11 (52.4) | 19 (41.3) |
| Bacterial sepsis | 1 (4.0) | 0 | 1 (2.2) |
| Sepsis | 7 (28.0) | 11 (52.4) | 18 (39.1) |
| Urinary tract infection | 5 (20.0) | 4 (19.0) | 9 (19.6) |
| Escherichia urinary tract infection | 1 (4.0) | 0 | 1 (2.2) |
| Urinary tract infection | 3 (12.0) | 4 (19.0) | 7 (15.2) |
| Urinary tract infection bacterial | 1 (4.0) | 0 | 1 (2.2) |
| Median (range) duration since onset, days | 4.0 (1, 11) | 2.0 (1, 23) | 2.0 (1, 23) |

^a^Age = date of screening – date of birth + 1.

^b^Corrected age for preterm infants in Part A, Cohort 1 (*n* = 2). Corrected age = age (days) + gestational age (days) – 280.

^c^Inclusion age is defined as the chronological age for term infants and the corrected age for preterm infants.

^d^Race and ethnicity data as reported by parents/legal representatives of the of the patient.

Cohort 1: Full-term infants with chronological age > 28 days to < 3 months or preterm infants with corrected age > 28 days to < 3 months.
Cohort 2: Full-term neonates with chronological age from birth to ≤ 28 days.
Cohort 3: Preterm neonates from birth to chronological age ≤ 28 days.

Table S2. Incidence of AEs as Reported by Investigators, up to LFU (Safety Analysis Set)

| **Patients, *n* (%)** | **Part A (*n*= 25)** | **Part B (*n*= 21)** | **Overall (*N*= 46)** |
| --- | --- | --- | --- |
| Any AE | 8 (32.0) | 15 (71.4) | 23 (50.0) |
| Blood and lymphatic system disorders | 1 (4.0) | 5 (23.8) | 6 (13.0) |
| Anemia | 0 | 3 (14.3) | 3 (6.5) |
| Anemia neonatal | 1 (4.0) | 0 | 1 (2.2) |
| Neutropenia | 0 | 2 (9.5) | 2 (4.3) |
| Cardiac disorders | 2 (8.0) | 1 (4.8) | 3 (6.5) |
| Atrial tachycardia | 1 (4.0) | 0 | 1 (2.2) |
| Atrial thrombosis | 1 (4.0) | 0 | 1 (2.2) |
| Cardiac failure acute | 0 | 1 (4.8) | 1 (2.2) |
| Pericardial effusion | 1 (4.0) | 0 | 1 (2.2) |
| Eye disorders | 0 | 1 (4.8) | 1 (2.2) |
| Retinopathy of prematurity | 0 | 1 (4.8) | 1 (2.2) |
| Gastrointestinal disorders | 2 (8.0) | 4 (19.0) | 6 (13.0) |
| Diarrhea | 0 | 1 (4.8) | 1 (2.2) |
| Necrotizing colitis | 1 (4.0) | 1 (4.8) | 2 (4.3) |
| Vomiting | 1 (4.0) | 2 (9.5) | 3 (6.5) |
| General disorders and administration site conditions | 1 (4.0) | 2 (9.5) | 3 (6.5) |
| Influenza-like illness | 0 | 1 (4.8) | 1 (2.2) |
| Pyrexia | 1 (4.0) | 1 (4.8) | 2 (4.3) |
| Infections and infestations | 2 (8.0) | 10 (47.6) | 12 (26.1) |
| COVID-19 | 1 (4.0) | 1 (4.8) | 2 (4.3) |
| Candida sepsis | 0 | 1 (4.8) | 1 (2.2) |
| Enterobacter sepsis | 0 | 1 (4.8) | 1 (2.2) |
| Enterococcal sepsis | 0 | 1 (4.8) | 1 (2.2) |
| Infection | 0 | 1 (4.8) | 1 (2.2) |
| Oral candidiasis | 1 (4.0) | 0 | 1 (2.2) |
| Pneumonia aspiration | 0 | 1 (4.8) | 1 (2.2) |
| Postoperative wound infection | 0 | 1 (4.8) | 1 (2.2) |
| Rhinovirus infection | 0 | 1 (4.8) | 1 (2.2) |
| Sepsis | 0 | 5 (23.8) | 5 (10.9) |
| Septic shock | 0 | 1 (4.8) | 1 (2.2) |
| Investigations | 4 (16.0) | 6 (28.6) | 10 (21.7) |
| ALT increased | 1 (4.0) | 0 | 1 (2.2) |
| Blood potassium increased | 0 | 1 (4.8) | 1 (2.2) |
| Blood thyroid stimulating hormone increased | 1 (4.0) | 0 | 1 (2.2) |
| Cardiac murmur | 1 (4.0) | 0 | 1 (2.2) |
| ECG QT prolonged | 1 (4.0) | 1 (4.8) | 2 (4.3) |
| Hepatic enzyme increased | 1 (4.0) | 1 (4.8) | 2 (4.3) |
| International normalized ratio increased | 1 (4.0) | 0 | 1 (2.2) |
| Oxygen saturation decreased | 1 (4.0) | 1 (4.8) | 2 (4.3) |
| Transaminases increased | 0 | 2 (9.5) | 2 (4.3) |
| Metabolism and nutrition disorders | 3 (12.0) | 1 (4.8) | 4 (8.7) |
| Fluid retention | 1 (4.0) | 0 | 1 (2.2) |
| Hypercholesterolemia | 0 | 1 (4.8) | 1 (2.2) |
| Hypermagnesemia | 1 (4.0) | 0 | 1 (2.2) |
| Hypertriglyceridemia | 0 | 1 (4.8) | 1 (2.2) |
| Hypoalbuminemia | 1 (4.0) | 0 | 1 (2.2) |
| Hyponatremia | 1 (4.0) | 0 | 1 (2.2) |
| Musculoskeletal and connective tissue disorders | 1 (4.0) | 0 | 1 (2.2) |
| Growth retardation | 1 (4.0) | 0 | 1 (2.2) |
| Nervous system disorders | 0 | 1 (4.8) | 1 (2.2) |
| Clonus | 0 | 1 (4.8) | 1 (2.2) |
| Psychiatric disorders | 1 (4.0) | 0 | 1 (2.2) |
| Delerium | 1 (4.0) | 0 | 1 (2.2) |
| Renal and urinary disorders | 2 (8.0) | 2 (9.5) | 4 (8.7) |
| Hydronephrosis | 1 (4.0) | 0 | 1 (2.2) |
| Oliguria | 1 (4.0) | 1 (4.8) | 2 (4.3) |
| Renal failure | 0 | 1 (4.8) | 1 (2.2) |
| Respiratory, thoracic, and mediastinal disorders | 1 (4.0) | 2 (9.5) | 3 (6.5) |
| Acute respiratory failure | 0 | 1 (4.8) | 1 (2.2) |
| Bronchomalacia | 1 (4.0) | 0 | 1 (2.2) |
| Bronchopulmonary dysplasia | 0 | 1 (4.8) | 1 (2.2) |
| Pneumothorax | 0 | 1 (4.8) | 1 (2.2) |
| Skin and subcutaneous tissue disorders | 1 (4.0) | 3 (14.3) | 4 (8.7) |
| Decubitus ulcer | 0 | 2 (9.5) | 2 (4.3) |
| Dermatitis diaper | 0 | 1 (4.8) | 1 (2.2) |
| Erythema | 1 (4.0) | 0 | 1 (2.2) |
| Vascular disorders | 0 | 1 (4.8) | 1 (2.2) |
| Hypertension | 0 | 1 (4.8) | 1 (2.2) |

Percentages are based on the total number of patients in each specified characteristic and total (N). patients are only counted once per treatment per event and include data up to LFU. Totals for the number of patients at a higher level are not necessarily the sum of those at the lower levels since a patient may report 2 or more different AEs within the higher-level category.

MedDRA v25.1 coding dictionary applied.

Abbreviations: AE, adverse event; ECG, echocardiogram; LFU, late follow-up; MedDRA, Medical Dictionary for Regulatory Activities.

Table S3. Incidence of SAEs as Reported by Investigators, up to LFU (Safety Analysis Set)

| **Patients, *n* (%)** | **Part A (*n*= 25)** | **Part B (*n*= 21)** | **Overall (*N*= 46)** |
| --- | --- | --- | --- |
| Blood and lymphatic system disorders | 0 | 1 (4.8) | 1 (2.2) |
| Neutropenia neonatal | 0 | 1 (4.8) | 1 (2.2) |
| Cardiac disorders | 0 | 1 (4.8) | 1 (2.2) |
| Cardiac failure acute | 0 | 1 (4.8) | 1 (2.2) |
| Congenital, familial, and genetic disorders | 0 | 1 (4.8) | 1 (2.2) |
| Gene mutation | 0 | 1 (4.8) | 1 (2.2) |
| Gastrointestinal disorders | 1 (4.0) | 1 (4.8) | 2 (4.3) |
| Necrotizing colitis | 1 (4.0) | 1 (4.8) | 2 (4.3) |
| General disorders and administration site conditions | 0 | 1 (4.8) | 1 (2.2) |
| Condition aggravated | 0 | 1 (4.8) | 1 (2.2) |
| Infections and infestations | 1 (4.0) | 4 (19.0) | 5 (10.9) |
| COVID-19 | 1 (4.0) | 0 | 1 (2.2) |
| Candida sepsis | 0 | 1 (4.8) | 1 (2.2) |
| Enterobacter sepsis | 0 | 1 (4.8) | 1 (2.2) |
| Enterococcal sepsis | 0 | 1 (4.8) | 1 (2.2) |
| Sepsis | 0 | 1 (4.8) | 1 (2.2) |
| Sepsis neonatal | 0 | 1 (4.8) | 1 (2.2) |
| Septic shock | 0 | 1 (4.8) | 1 (2.2) |
| Investigations | 1 (4.0) | 1 (4.8) | 2 (4.3) |
| ECG QT prolonged | 0 | 1 (4.8) | 1 (2.2) |
| Hepatic enzyme increased | 1 (4.0) | 0 | 1 (2.2) |
| Renal and urinary disorders | 0 | 2 (9.5) | 2 (4.3) |
| Oliguria | 0 | 1 (4.8) | 1 (2.2) |
| Renal failure | 0 | 1 (4.8) | 1 (2.2) |
| Respiratory, thoracic, and mediastinal disorders | 0 | 1 (4.8) | 1 (2.2) |
| Neonatal respiratory failure | 0 | 1 (4.8) | 1 (2.2) |
| Total preferred term events | 3 | 15 | 18 |
| Total number of cases | 3 | 10 | 13 |
| Total number of patients with SAEs | 3 | 5 | 8 |

Percentages are based on the total number of patients in each specified characteristic and total (N). patients are only counted once per treatment per event and include data up to LFU. Totals for the number of patients at a higher level are not necessarily the sum of those at the lower levels since a patient may report 2 or more different AEs within the higher-level category.

MedDRA v25.1 coding dictionary applied.

Abbreviations: AE, adverse event; ECG, echocardiogram; LFU, late follow-up; MedDRA, Medical Dictionary for Regulatory Activities; SAE, serious adverse events.

Table S4. Plasma Concentrations (µg/mL) of Ceftazidime by Nominal Time Post Dose Excluding Patient with Medication Error (Pharmacokinetic Analysis Set)

| **Time point** | **Statistics** | **Part A** | | | | **Part B** | | | |
| --- | --- | --- | --- | --- | --- | --- | --- | --- | --- |
|  |  | **Cohort 1 (*n* = 8)** | **Cohort 2 (*n* = 8)** | **Cohort 3 (*n* = 8)** | **Total (*N* = 24)** | **Cohort 1 (*n* = 8)** | **Cohort 2 (*n* = 4)** | **Cohort 3 (*n*= 8)** | **Total (*N* = 20)** |
| 2 h | *n* | 8 | 8 | 8 | 24 | 8 | 4 | 8 | 20 |
|  | Mean (SD) | 67.2 (17.9) | 35.6 (13.5) | 53.2 (26.0) | 52.0 (23.1) | 53.0 (17.6) | 48.6 (17.0) | 43.7 (22.2) | 48.4 (19.0) |
|  | Median (min, max) | 61.2 (47.7, 95.3) | 33.9 (17.3, 53.3) | 41.4 (35.0, 113.0) | 49.2 (17.3, 113.0) | 49.7 (27.5, 77.6) | 55.6 (23.3, 60.0) | 49.3 (3.8, 73.8) | 51.2 (3.8, 77.6) |
|  | Q1, Q3 | 52.7, 83.7 | 25.1, 47.9 | 38.7, 58.8 | 36.1, 61.0 | 41.1, 68.5 | 39.4, 57.9 | 32.0, 54.8 | 41.1, 58.7 |
|  | CV, % | 26.6 | 37.9 | 48.8 | 44.4 | 33.2 | 35.0 | 50.9 | 39.2 |
|  | Geometric mean | 65.2 | 33.1 | 49.3 | 47.4 | 50.3 | 45.6 | 33.8 | 42.1 |
| 2 h 30 min | *n* | 8 | 7 | 8 | 23 | 8 | 4 | 8 | 20 |
|  | Mean (SD) | 41.7 (15.4) | 32.6 (10.8) | 49.0 (18.8) | 41.5 (16.3) | 43.0 (17.4) | 39.0 (4.4) | 42.5 (26.8) | 42.0 (19.5) |
|  | Median (min, max) | 37.1 (27.9, 66.9) | 29.4 (18.1, 50.0) | 42.2 (32.2, 89.5) | 37.8 (18.1, 89.5) | 43.9 (18.4, 66.0) | 41.0 (32.4, 41.7) | 45.2 (23.2, 94.6) | 42.0 (23.2, 94.6) |
|  | Q1, Q3 | 29.4, 52.0 | 26.0, 42.9 | 36.5, 56.6 | 29.4, 50.0 | 28.5, 57.6 | 36.6, 41.4 | 29.7, 46.6 | 34.5, 46.7 |
|  | CV, % | 37.0 | 33.3 | 38.4 | 39.4 | 40.5 | 11.3 | 63.1 | 46.5 |
|  | Geometric mean | 39.4 | 31.0 | 46.4 | 38.8 | 39.4 | 38.8 | 30.2 | 35.3 |
| 7 h | *n* | 8 | 8 | 8 | 24 | 8 | 4 | 8 | 20 |
|  | Mean (SD) | 11.1 (7.2) | 8.3 (6.7) | 17.6 (8.2) | 12.3 (8.1) | 8.3 (4.8) | 16.7 (9.1) | 18.7 (18.3) | 14.1 (13.0) |
|  | Median (min, max) | 8.4 (0.5, 23.6) | 9.2 (1.0, 21.9) | 15.8 (7.7, 35.3) | 9.8 (1.0, 35.3) | 8.5 (1.3, 15.0) | 14.7 (9.4, 28.1) | 14.4 (1.0, 59.8) | 9.8 (1.0, 59.8) |
|  | Q1, Q3 | 6.1, 15.5 | 2.4, 10.2 | 13.6, 19.6 | 7.2, 17.0 | 4.8, 11.9 | 9.4, 241. | 7.9, 22.0 | 7.0, 19.3 |
|  | CV, % | 65.0 | 81.0 | 46.4 | 65.8 | 57.7 | 54.2 | 97.9 | 91.9 |
|  | Geometric mean | 9.5 | 5.7 | 16.2 | 9.6 | 6.5 | 14.9 | 11.6 | 9.6 |

Cohort 1: Full-term infants with chronological age > 28 days to < 3 months or preterm infants with corrected age > 28 days to < 3 months.
Cohort 2: Full-term neonates with chronological age from birth to ≤ 28 days.
Cohort 3: Preterm neonates from birth to chronological age ≤ 28 days.

Abbreviations: CV, coefficient of variation; Q, quartile; SD, standard deviation.

Table S5. Plasma Concentrations (µg/mL) of Avibactam by Nominal Time Post Dose Excluding Patient With Medication Error (Pharmacokinetic Analysis Set)

| **Time point** | **Statistics** | **Part A** | | | | **Part B** | | | |
| --- | --- | --- | --- | --- | --- | --- | --- | --- | --- |
|  |  | **Cohort 1 (*n* = 8)** | **Cohort 2 (*n* = 8)** | **Cohort 3 (*n* = 8)** | **Total (*N* = 24)** | **Cohort 1 (*n* = 8)** | **Cohort 2 (*n* = 4)** | **Cohort 3 (*n* = 8)** | **Total (*n* = 20)** |
| 2 h | *n* | 8 | 8 | 8 | 24 | 8 | 4 | 8 | 20 |
|  | Mean (SD) | 13.1 (3.8) | 6.9 (2.5) | 10.6 (4.3) | 10.2 (4.3) | 10.3 (3.3) | 11.3 (2.0) | 9.4 (5.6) | 10.2 (4.1) |
|  | Median (min, max) | 12.0 (9.3, 19.6) | 6.8 (3.0, 10.1) | 9.0 (6.5, 20.5) | 9.3 (3.0, 20.5) | 10.4 (4.6, 14.8) | 10.6 (9.9, 14.3) | 9.0 (0.5, 19.0) | 10.4 (0.5, 19.0) |
|  | Q1, Q3 | 9.8, 16.1 | 5.1, 9.1 | 8.6, 11.3 | 7.9, 12.0 | 8.6, 12.8 | 10.1, 12.6 | 6.0, 12.0 | 8.6, 12.8 |
|  | CV, % | 29.0 | 37.0 | 41.1 | 42.6 | 31.6 | 17.8 | 59.0 | 39.9 |
|  | Geometric mean | 12.6 | 6.4 | 10.0 | 9.3 | 9.8 | 11.2 | 6.8 | 8.7 |
| 2 h 30 min | *n* | 8 | 7 | 8 | 23 | 8 | 4 | 8 | 20 |
|  | Mean (SD) | 7.7 (3.0) | 6.3 (2.4) | 9.8 (3.0) | 8.0 (3.1) | 6.8 (2.4) | 9.4 (2.3) | 9.1 (5.7) | 8.2 (4.0) |
|  | Median (min, max) | 6.9 (3.7, 12.6) | 5.5 (3.2, 10.6) | 9.7 (5.3, 15.0) | 7.4 (3.2, 15.0) | 6.9 (3.3, 9.9) | 8.8 (7.4, 12.6) | 9.9 (0.5, 19.1) | 8.4 (0.5, 19.1) |
|  | Q1, Q3 | 5.8, 9.9 | 5.0, 7.4 | 8.0, 11.4 | 5.3, 10.6 | 4.9, 8.9 | 7.7, 11.0 | 5.0, 11.6 | 5.7, 10.0 |
|  | CV, % | 39.3 | 37.8 | 30.2 | 38.4 | 35.0 | 24.7 | 62.8 | 49.0 |
|  | Geometric mean | 7.2 | 5.9 | 9.4 | 7.4 | 6.4 | 9.2 | 6.4 | 6.9 |
| 7 h | n | 8 | 8 | 8 | 24 | 8 | 4 | 8 | 20 |
|  | Mean (SD) | 1.7 (1.4) | 1.2 (1.0) | 3.5 (1.9) | 2.1 (1.7) | 1.0 (0.6) | 3.8 (2.5) | 3.9 (3.9) | 2.7 (2.9) |
|  | Median (min, max) | 1.1 (0.6, 4.4) | 1.2 (0.2, 3.3) | 3.4 (0.8, 6.4) | 1.3 (0.2, 6.4) | 8.2  0.4, 1.8) | 3.5 (1.0, 7.1) | 3.0 (0.1, 12.4) | 1.7 (0.1, 12.4) |
|  | Q1, Q3 | 0.9, 2.4 | 0.3, 1.5 | 2.1, 4.9 | 0.9, 3.4 | 0.6, 1.6 | 2.0, 5.6 | 1.4, 4.9 | 0.8, 4.0 |
|  | CV, % | 81.9 | 83.9 | 55.6 | 81.9 | 57.8 | 66.9 | 99.4 | 108.1 |
|  | Geometric mean | 1.3 | 0.8 | 2.9 | 1.5 | 0.9 | 3.1 | 2.2 | 1.6 |

Cohort 1: Full-term infants with chronological age > 28 days to < 3 months or preterm infants with corrected age > 28 days to < 3 months.
Cohort 2: Full-term neonates with chronological age from birth to ≤ 28 days.
Cohort 3: Preterm neonates from birth to chronological age ≤ 28 days.

Abbreviations: CV, coefficient of variation; Q, quartile; SD, standard deviation.

Table S6. Clinical Outcomes by Study Visit (Part B; MITT Analysis Set)

| ***n* (%)** | **Cohort 1 (*n* = 8)** | **Cohort 2 (*n* = 3)** | **Cohort 1 (*n* = 5)** | **Overall (*N* = 16)** |
| --- | --- | --- | --- | --- |
| EOIV |  |  |  |  |
| Favorable outcome | 8 (100) | 2 (66.7) | 3 (60.0) | 13 (81.3) |
| Clinical cure | 2 (25.0) | 1 (33.3) | 3 (60.0) | 6 (37.5) |
| Clinical improvement | 6 (75.0) | 1 (33.3) | 0 | 7 (43.8) |
| Clinical failure | 0 | 0 | 1 (20.0) | 1 (6.3) |
| Indeterminate | 0 | 1 (33.3) | 1 (20.0) | 2 (12.5) |
| Missing | 0 | 0 | 0 | 0 |
| EOT |  |  |  |  |
| Favorable outcome | 8 (100) | 2 (66.7) | 3 (60.0) | 13 (81.3) |
| Clinical cure | 6 (75.0) | 1 (33.3) | 3 (60.0) | 10 (62.5) |
| Clinical improvement | 2 (25.0) | 1 (33.3) | 0 | 3 (18.8) |
| Clinical failure | 0 | 0 | 1 (20.0) | 1 (6.3) |
| Indeterminate | 0 | 1 (33.3) | 0 | 1 (6.3) |
| Missing | 0 | 0 | 1 (20.0) | 1 (6.3) |
| TOC |  |  |  |  |
| Favorable outcome | 7 (87.5) | 1 (33.3) | 4 (80.0) | 12 (75.0) |
| Clinical cure | 7 (87.5) | 1 (33.3) | 4 (80.0) | 12 (75.0) |
| Clinical improvement | 0 | 0 | 0 | 0 |
| Clinical failure | 0 | 0 | 1 (20.0) | 1 (6.3) |
| Indeterminate | 0 | 2 (66.7) | 0 | 2 (12.5) |
| Missing | 0 | 0 | 0 | 0 |
| LFU |  |  |  |  |
| Favorable outcome | 8 (100) | 2 (66.7) | 3 (60.0) | 13 (81.3) |
| Clinical cure | 8 (100) | 2 (66.7) | 3 (60.0) | 13 (81.3) |
| Clinical improvement | 0 | 0 | 0 | 0 |
| Clinical failure | 0 | 0 | 1 (20.0) | 1 (6.3) |
| Indeterminate | 0 | 1 (33.3) | 0 | 1 (6.3) |
| Missing | 0 | 0 | 0 | 0 |

Cohort 1: Full-term infants with chronological age > 28 days to < 3 months or preterm infants with corrected age > 28 days to < 3 months.
Cohort 2: Full-term neonates with chronological age from birth to ≤ 28 days.
Cohort 3: Preterm neonates from birth to chronological age ≤ 28 days.

Abbreviations: EOIV, end of intravenous treatment; EOT, end of treatment; LFU, late follow-up; MITT, modified intent-to-treat; TOC, test of care.

# Reference

1. Engle WA, American Academy of Pediatrics Committee on Fetus and Newborn. Age terminology during the perinatal period. Pediatrics **2004**; 114:1362–1364.
